# On the Physical Meaning of the Isothermal Titration Calorimetry Measurements in Calorimeters with Full Cells

**DOI:** 10.3390/ijms10125296

**Published:** 2009-12-09

**Authors:** Jean-Pierre E. Grolier, Jose Manuel del Río

**Affiliations:** 1 Laboratoire de Thermodynamique des Solutions et des Polymères Université Blaise Pascal, 24, Avenue des Landais, Clermont Ferrand, 63177 Aubière Cedex, France; E-Mail: j-pierre.grolier@univ-bpclermont.fr (J.-P.E.G.); 2 Research and Development Branch on Flow Assurance, Mexican Institute of Petroleum, Eje Central Lazaro Cardenas 152, Col. San Bartolo Atepehuacan, Mexico City 07730, Mexico

**Keywords:** isothermal titration calorimetry, ITC, calorimetry, thermodynamics, infinite dilution, binding, partial properties, enthalpy, heat

## Abstract

We have performed a detailed study of the thermodynamics of the titration process in an isothermal titration calorimeter with full cells. We show that the relationship between the enthalpy and the heat measured is better described in terms of the equation Δ H = W_inj_ + Q (where W_inj_ is the work necessary to carry out the titration) than in terms of ΔH = Q. Moreover, we show that the heat of interaction between two components is related to the partial enthalpy of interaction at infinite dilution of the titrant component, as well as to its partial volume of interaction at infinite dilution.

## 1. Introduction

Isothermal titration calorimetry [1] is a fundamental quantitative biochemical tool for characterizing intermolecular interactions, such as protein-ligand, protein-protein, drug-DNA and protein-DNA. It uses stepwise injections of one reagent into a calorimetric cell containing the second reagent to measure the heat of the reaction for both exothermic and endothermic processes.

**Figure 1.**
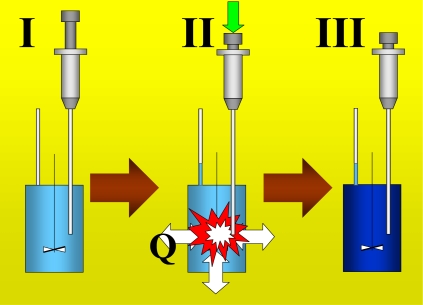
Typical performance of an isothermal titration calorimeter. The electronic details of the measurement of the calorimetric signal have been omitted for clarity.

Figure 1 shows the basic performance of a titration in an isothermal titration calorimeter with full cells. The titration cell (I) is composed of a vessel, a syringe containing a second liquid and a drainage capillary, through which liquid in excess is removed from the full cell upon introduction of a new liquid from the syringe. The vessel is maintained at a constant temperature, and the interior liquid is stirred to achieve homogeneity.

When the liquid of the vessel interior (see Figure 1-I) is titrated with the amount of liquid in the syringe heat flows from or to the vessel (Figure 1-II); this heat flow is measured and recorded by a suitable electronic system. At this time, a volume of liquid equal to that of the titrant liquid exits the vessel through the drainage capillary (Figure 1-II). In the final state (1-III), the interior of the vessel contains the two liquids that are completely mixed at a known composition and the drainage capillary holds an amount of liquid with a different composition. Thus, it is possible to consider an effective volume in the vessel in which a determinate amount of heat is produced (or adsorbed) and in which the concentrations are known. Importantly, this effective volume is constant throughout the titration process. If the cell is half-full, however, this assumption is not necessarily correct, because the volume of sample varies in the process of titration. In this work, we consider only full cell titration calorimeters. Table 1 shows a list of isothermal titration calorimeters that are currently commercially available. The majority of these calorimeters use the full cells method.

It is commonly accepted that with a suitable procedure involving simple titration experiments [2], it is possible to measure the heat of interaction between two components (components 2 and 3) in a solvent (component 1). In a first experiment, a solution of component 2 in the solvent (component 1) is titrated with a stock solution of component 3 in the same solvent. The contributions to the heat that is measured are the heat of interaction between the components 2 and 3, the heats of dilution of components 2 and 3, and the heats of interaction between the component 3 and the different parts of the experimental setup (vessel walls, stirrer and syringe needle). In a second experiment the solvent (component 1) is titrated with the stock solution.

**Figure 2.**
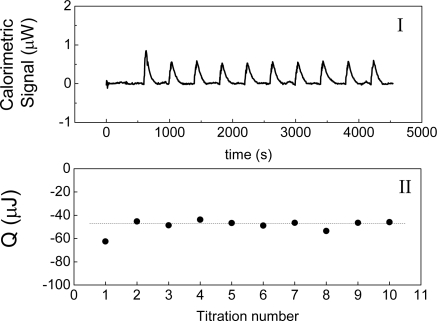
Titration of water with water at 30 °C. Graph I shows the calorimetric signal as function of the time and graph II shows the heat involved in each titration. This heat is calculated by the integral of the calorimetric signal between the initial and final times for each peak. The volume titrated for each peak is 20 μL and the volume cell is 1,300 μL.

This experiment is carried out using the same conditions as the first experiment. In this case, the contributions to the heat measured are the heat of dilution of the component 3 and the interaction with the different parts of the experimental setup. In the third experiment the solution of component 2 in the solvent is titrated with the solvent and the heat of dilution of component 2 is the contribution to the heat measured. In the fourth experiment the solvent is titrated with the solvent. Figure 2 shows an example of this experiment, in which water is titrated with water. The heat of interaction is interpreted as the following balance:

(1) $$\begin{array}{ll} \text{Heat}\;\text{of}\;\text{interaction}= & [\text{Heat}\;\text{of}\;\text{experiment}\;1]-[\text{Heat}\;\text{of}\;\text{experiment}\;2]-\\ & -[\text{Heat}\;\text{of}\;\text{experiment}\;3]\;+\;[\text{Heat}\;\text{of}\;\text{experiment}\;4] \end{array}$$

The fourth experiment takes part in the protocol because its contribution appears also in experiments one, second and three. From a practical point of view, the heats of experiments 3 and 4 are negligible since they are usually insignificant [2]. In this way, Equation (1) takes the form [1,2]:

(2) Heat of interaction = [Heat of experiment 1] − [Heat of experiment 2]

Because all processes in the above protocol are carried out at constant pressure, the measured heat is usually interpreted in terms of the following Equation [3]:

(3) Q = ΔH

where ΔH is the difference in enthalpy between the final and the initial estates, and Q is the heat measured by the calorimeter. The heat of interaction that is obtained from the above protocol is usually interpreted as the enthalpy of interaction.

It is interesting to note that the origin of the protocol shown in Equations (1) and (2) is empirical. The use of the set of Equations (1) and (2) to obtain heats of interaction seems reasonable and reliable, and it is supported by a considerable amount of experimental evidence; nonetheless, we do not have a rigorous demonstration that this heat can be considered as a heat of interaction. Thus, we do not know if this interpretation is exact or if it is an approximation. If it is an approximation, it would be useful to know under what conditions it can be applied. It is also very interesting to note that Equation (3) is inconsistent with the considerations made in the protocol shown in the Equations (1) and (2). If for example, we consider the titration of water in water, the initial state is a volume V of pure water, and the final state after titration is this volume V of pure water. The difference in enthalpy for this system is zero. From Equation (3), the expected heat for this experiment is zero, against the experimental result shown in Figure 2. Without the Equation (3) the problem now is the following: can we interpret the heats obtained by Equations (1) and (2) as enthalpies of interaction?

In this paper, we address the above problem and the physical meaning of the heat obtained from the given protocol based on the typical performance of an isothermal titration with full cells which is described in Figure 1. We first aimed to find a new equation to replace the equation Q = ΔH [Equation (3)]. Next, we determined how the concentrations of different components vary after the titration. Then, we calculated the heats involved in the titration process. We also applied a set of thermodynamic tools that were developed in our previous works [4–6]. We consider the hypothesis that the solutions are sufficiently diluted. This hypothesis was mathematically implemented, supposing that the molar (or specific) thermodynamic properties could be described by a Taylor expansion of the first order (high diluted region). Another concept that we applied is the “fraction of a system”. A fraction of a system is a thermodynamic entity (with internal composition) that groups several components. This concept is essential for working with multicomponent systems at infinite dilution.

We observed that the heat measured in an experiment where solvent is titrated with itself has its origin in the work required for to inject the volume of titrant. For this reason it can be named as “heat of injection”. In addition, we see that the heat involved per mol of titrant when the titration is infinitesimally small is related to its partial molar enthalpy of interaction at infinite dilution and its molar partial volume of interaction also at infinite dilution. That is, using the full-cell method, the heat measured by the calorimeter when the above protocol is employed is the partial molar enthalpy of interaction only when the variation in the molar partial volume of interaction can be neglected. This fact is true in binding events where protein unfolding is involved.

## 2. Experimental

The calorimeter used was an ITC 4200 from CSC equipped to work with nanowatt sensitivity. The volume cell is 1,300 μL. The working temperature was in all cases 30 °C. The water used was bidestilated and the toluene (reagent grade) was obtained from Fermont.

## 3. Thermodynamics

### 3.1. Application of the First Principle of Thermodynamics to the titration process with constant P and V

From a thermodynamic point of view, the process of titration shown in Figure 1 can be described as a process in which the temperature T, the pressure P and the volume V are kept constant. Applying the First Principle of Thermodynamics to this titration process we have:

(4) ΔU = Q + W

where ΔU is the difference in internal energy of the system inside a cell with volume V, Q is the heat measured by the calorimeter and W is the work, which we need to bear in mind when we are considering the First Principle of Thermodynamics. Because the enthalpy is the Legendre transform of the internal energy U, it is possible to write:

(5) H = U + PV

Thus, for a process in which the pressure and the volume are maintained constant, the variation in the enthalpy is:

(6) ΔH = ΔU

Substituting Equation (6) into Equation (4) we have:

(7) ΔH = Q + W

**Figure 3.**
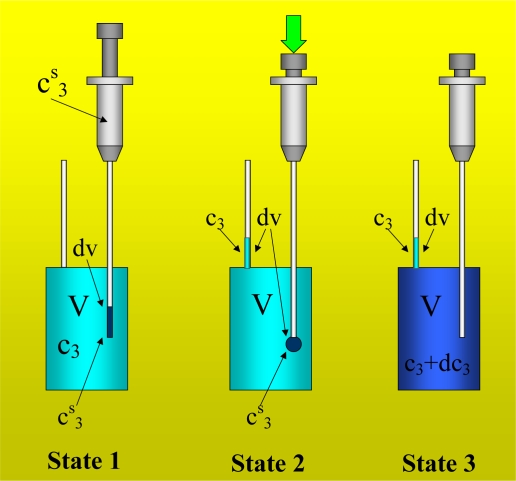
Different states to be considered during the titration process for an experiment of concentration of component 3. The first state (State 1) is a volume V (vessel volume) of solution with concentration c_3_. The concentration of component 3 in the syringe is c^s^_3_. This state also includes a volume dv of stock solution with a concentration c^s^_3_ at the end of the needle before the titration. In the second state (State 2), the volume dv of stock solution is introduced into the volume of the vessel while a volume dv with concentration c_3_ exits from the vessel volume by the drainage capillary. In the third state (State 3), the composition of the vessel interior is homogenized until it achieves the new concentration c_3_ + dc_3_; the drainage capillary includes a volume dv of solution with concentration c_3_.

With Equation (7) it is possible to explain the calorimetric signal that is obtained when a liquid is titrated with itself. As noted in the “Introduction”, ΔH = 0 for this process, substituting this result into (7) yields Q = -W. That is, the amount of heat obtained comes from the work performed. This work is very easy to identify. In Figure 3 (State 2) we show that the titration is carried out by the displacement of the syringe plunger, which introduces an amount of liquid into the vessel and forces the exit of the same amount of liquid through the drainage capillary. Thus, it is necessary to apply work to replace an amount of liquid in the vessel. This work, W_inj_, will be named “injection work”; and the heat measured by the calorimeter is then:

(8) $${\text{Q}}_{\text{inj}}\;=\;-{\text{W}}_{\text{inj}}$$

and will be named “injection heat”. Therefore the application of the First Principle of thermodynamics to the general titration process [Equation (7)] takes the form:

(9) $$\Delta \text{H}\;=\;{\text{W}}_{\text{inj}}\;+\;\text{Q}$$

Note that in Equation (9) the enthalpy variation results from the contribution of heat (measured by the calorimeter) and the work of titration. In addition, through Equation (6), this variation in internal energy is derived directly from the variation in enthalpy.

### 3.2. Determination of the concentrations in the process of titration

In this section, we will determinate the concentrations in experiments where a solution of components 2 and 3 in a solvent (component 1) is titrated with a stock solution of 3 in the same solvent. This titration experiment can be described as the combination of two simpler experiments. The first experiment that we will address is one in which a solution of component 3 in a solvent is titrated with a more concentrated stock solution of component 3 in the same solvent. Because the concentration of component 3 will increase in each titration, this type of experiment will be named “concentration experiment”. The other experiment is one in which a solution of component 2 in a solvent is titrated only with the solvent. In this case, the concentration of component 2 will decrease with each titration; for this reason this experiment will be named “dilution experiment”. The more complex experiment, in which a solution of components 2 and 3 in a solvent is titrated with a more concentrated stock solution of 3 in the same solvent, can be considered to be the combination of two simultaneous experiments: a dilution experiment component 2 and a concentration experiment for component 3. This experiment will named the “concentration-dilution experiment”.

The concentrations of 2 and 3 in component 1 are expressed as c_2_ = n_2_/V and c_3_ = n_3_/V, with n_2_ and n_3_ being the numbers of moles of components 2 and 3, respectively.

#### 3.2.1. Concentration experiment in 2-component systems

Let us now consider the system in Figure 3. In State 1, a solution of component 3 in component 1 is located in the vessel at an initial concentration c^(i)^_3_; and in the syringe, is present as a stock solution with a concentration c^s^_3_. We will consider the infinitesimal process with respect to the titration volume in which the solution of the vessel with concentration c_3_ is titrated with a volume dv of stock solution. The different steps of this infinitesimal process are shown in Figure 3.

In the first state, the number of moles of component 3, n_3_, in the volume V is:

(10) $${\text{n}}_{3}\;=\;{\text{c}}_{3}\text{V}$$

This solution (see State 1 of Figure 3) will be titrated with a volume dv of stock solution of concentration c^s^_3_. The number of moles of component 3 contained in the volume dv is:

(11) $${\text{dn}}_{3}^{\text{s}}\;=\;{\text{c}}_{3}^{\text{s}}\text{dv}$$

In State 2, the volume dv of stock solution is introduced into the vessel. Because the volume of the vessel is constant, a similar volume with concentration c_3_ is removed from the vessel by the drainage capillary. The amount of moles of 3 that is pushed out is:

(12) $${\text{dn}}_{3}\;=\;{\text{c}}_{3}\text{dv}$$

In state 2 (see Figure 3), the interior of the vessel contains a volume V-dv with concentration c_3_ and another solution of volume dv with concentration c^s^_3_. In the State 3, the above solutions are mixed, and the new concentration inside the vessel is c_3_ + dc_3_, with (c_3_ + dc_3_)V being the final number of moles of component 3 in the vessel. Balancing the number of moles for the titration process, we have:

(13) $$({\text{c}}_{3}\;+\;{\text{dc}}_{3})\text{V}\;=\;{\text{n}}_{3}\;+\;{\text{dn}}_{3}^{\text{s}}\;-\;{\text{dn}}_{3}$$

where initially there were n_3_ moles of component 3, dn^s^_3_ moles were introduced into the vessel and dn_3_ moles were removed. Substituting the Equations (10)–(12) into (13) and reorganizing yields:

(14) $$\frac{{\text{dc}}_{3}}{\text{dv}}\;+\;\frac{1}{\text{V}}{\text{c}}_{3}\;-\;\frac{1}{\text{V}}{\text{c}}_{3}^{\text{s}}\;=\;0$$

Equation (14) is a linear differential equation of the first order, and its solution will be a function of v, c_3_ = c_3_(v), with the initial condition:

(15) $${\text{c}}_{3}^{\left(\text{i}\right)}\;=\;{\text{c}}_{3}(0)$$

then the solution c_3_ = c_3_(v) can be written as:

(16) $${\text{c}}_{3}(\text{v})={\text{c}}_{3}^{\text{s}}-({\text{c}}_{3}^{\text{s}}-{\text{c}}_{3}^{\left(\text{i}\right)}){\text{e}}^{-\frac{\text{v}}{\text{V}}}$$

#### 3.2.2. Dilution experiment in 2-component systems

In this experiment, we will consider that a solution of component 2 in component 1 is located in the vessel and that this solution is titrated with an amount of component 1. Assuming that there are similar states in this process as those presented in Figure 3, that there is a similar balance of number of moles as in Equation (13), and that c^s^_3_ = 0 because the syringe holds only component 1, we then obtain the equation:

(17) $${\text{c}}_{2}(\text{v})={\text{c}}_{2}^{\left(\text{i}\right)}{\text{e}}^{-\frac{\text{v}}{\text{V}}}$$

#### 3.2.3. Concentration-dilution experiment in 3-component systems

We consider the case in which the vessel contains a solution of components 2 and 3 in component 1 which is titrated with a solution of component 3 in component 1. The initial concentrations of 2 and 3 are c^(i)^_2_ and c^(i)^_3_ respectively. This experiment can be considered as the sum of two experiments: the dilution the component 2 and the concentration of component 3. In the first, the concentration of 2 after titration is given by Equation (17). In the second, the concentration of 3 after the titration is given by Equation (16). For convenience we define the variables c_F_ and t_f3_ as:

(18) $${\text{c}}_{\text{F}}(\text{v})={\text{c}}_{2}(\text{v})+{\text{c}}_{3}(\text{v})$$

and:

(19) $${\text{x}}_{\text{f}\;3}(\text{v})=\frac{{\text{c}}_{3}(\text{v})}{{\text{c}}_{2}(\text{v})+{\text{c}}_{3}(\text{v})}$$

Upon substituting (16) and (17) into Equations (18) and (19), we obtain:

(20) $${\text{c}}_{\text{F}}(\text{v})={\text{c}}_{3}^{\text{s}}-({\text{c}}_{3}^{\text{s}}-{\text{c}}_{\text{F}}^{\left(\text{i}\right)}){\text{e}}^{-\frac{\text{v}}{\text{V}}}$$

and:

(21) $${\text{x}}_{\text{f}3}(\text{v})=\frac{{\text{c}}_{3}^{\text{s}}-({\text{c}}_{3}^{\text{s}}-{\text{c}}_{3}^{\left(\text{i}\right)}){\text{e}}^{-\frac{\text{v}}{\text{V}}}}{{\text{c}}_{3}^{\text{s}}-({\text{c}}_{3}^{\text{s}}-{\text{c}}_{\text{F}}^{\left(\text{i}\right)}){\text{e}}^{-\frac{\text{v}}{\text{V}}}}$$

where c_F_^(i)^ = c_2_^(i)^ + c_3_^(i)^.

### 3.3. Determination of heats involved in the titration processes

In this section, we will determinate the heats that are involved in the different titration experiments: the concentration experiment, the dilution experiment and the concentration-dilution experiment. The heat of stirring (homogenization) is the same in all cases (all States). Then it cancels into the thermomechanical balance.

#### 3.3.1. Concentration experiment in 2-component systems

In State 1 of Figure 3, we have a solution of volume V and concentration c_3_ in the interior of the vessel; before the titration, a volume dv of solution stock with concentration c^s^_3_ is present at the end of the syringe needle. The enthalpy of the state 1, H_1_, is:

(22) $${\text{H}}_{1}=\text{H}({\text{c}}_{3},\text{V})+\text{H}({\text{c}}_{3}^{\text{s}},\text{dv})$$

In State 2 of Figure 3, inside the vessel we have a volume dv of stock solution with concentration c^s^_3_ and a volume V-dv of solution with concentration c_3_; outside the vessel, in the drainage capillary, we have a volume dv of concentration c_3_. The enthalpy of state 2, H_2_, is:

(23) $${\text{H}}_{2}=[\text{H}({\text{c}}_{3}^{\text{s}},\text{dv})+\text{H}({\text{c}}_{3},\text{V}-\text{dv})]+\text{H}({\text{c}}_{3},\text{dv})$$

In State 3 of Figure 3, the vessel contains a solution of concentration c_3_ + dc_3_ and the drainage capillary has a volume dv of concentration c_3_. The enthalpy of the state 3, H_3_, is:

(24) $${\text{H}}_{3}=\text{H}({\text{c}}_{3}+{\text{dc}}_{3},\text{V})+\text{H}({\text{c}}_{3},\text{dv})$$

**Figure 4.**
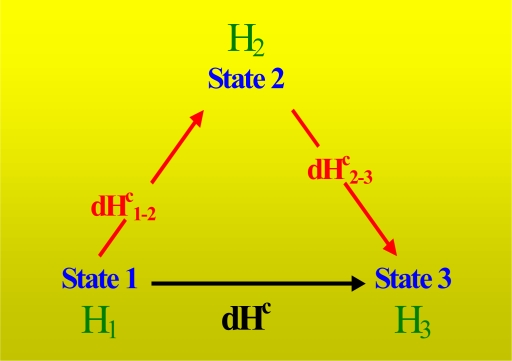
Variation in enthalpy between the different states of a differential concentration experiment of titration.

Figure 4 shows the variation in enthalpy between the different states of the titration process. The variation in enthalpy, dH^c^_1–2_, for the process 1–2 between states 1 and 2 is defined as:

(25) $${\text{dH}}_{1-2}^{\text{c}}={\text{H}}_{2}-{\text{H}}_{1}$$

and the variation in enthalpy, dH^c^_2–3_, for the process 2–3 between states 2 and 3 is:

(26) $${\text{dH}}_{2-3}^{\text{c}}={\text{H}}_{3}-{\text{H}}_{2}$$

The variation in enthalpy, dH^c^, for the entire process of titration between states 1 and 3 is:

(27) $${\text{dH}}^{\text{c}}={\text{H}}_{3}-{\text{H}}_{1}={\text{dH}}_{1-2}^{\text{c}}+{\text{dH}}_{2-3}^{\text{c}}$$

Applying the First Principle of Thermodynamics (Equation (9)) in the differential form to the process 1–2, we obtain:

(28) $${\text{dH}}_{1-2}^{\text{c}}={\text{dW}}_{1-2}^{\text{c}}+{\text{dQ}}_{1-2}^{\text{c}}$$

The value of dH^c^_1–2_ can be calculated by substituting the values of H_1_ and H_2_ (Equations (22) and (23)) for the definition of dH^c^_1–2_ (Equation (25)):

(29) $${\text{dH}}_{1-2}^{\text{c}}=\text{H}({\text{c}}_{3},\text{V}-\text{dv})+\text{H}({\text{c}}_{3},\text{dv})-\text{H}({\text{c}}_{3},\text{V})$$

Considering that H(c_3_,V) = h_v_(c_3_)V (Equation (153) in “Appendix 4: Basic equations”) one has:

(30) $$\begin{array}{l} \text{H}({\text{c}}_{3},\text{dv})={\text{h}}_{\text{v}}({\text{c}}_{3})\text{dv}\\ \text{H}({\text{c}}_{3},\text{V}-\text{dv})={\text{h}}_{\text{v}}({\text{c}}_{3})[\text{V}-\text{dv}]={\text{h}}_{\text{v}}({\text{c}}_{3})\text{V}-{\text{h}}_{\text{v}}({\text{c}}_{3})\text{dv} \end{array}$$

By substituting (30) into (29), we obtain the value of dH^c^_1–2_:

(31) $${\text{dH}}_{1-2}^{\text{c}}=0$$

The applying in that case the First Principle of Thermodynamics [Equation (9)] for the process 1–2 we have:

(32) $${\text{dQ}}_{1-2}^{\text{c}}=-{\text{dW}}_{1-2}^{\text{c}}$$

That is, the heat involved in the process 1–2, dQ^c^_1–2_, comes from the work applied in order to introduce a volume dv of stock solution into the interior of the vessel while an equal volume dv of solution with concentration c_3_ is pushed out from the vessel.

Applying the First Principle of Thermodynamics [Equation (9)] to the process 2–3 yields:

(33) $${\text{dH}}_{2-3}^{\text{c}}={\text{dW}}_{2-3}^{\text{c}}+{\text{dQ}}_{2-3}^{\text{c}}$$

In process 2–3, only a homogenizing process occurs in the vessel; thus, the work of injection is zero and:

(34) $${\text{dH}}_{2-3}^{\text{c}}={\text{dQ}}_{2-3}^{\text{c}}$$

This process of homogenizing involves the interaction between components 2 and 3. It is possible to calculate dH^c^_2–3_ by introducing the values of H_2_ and H_3_ (Equations (23) and (24)) into the definition of dH^c^_2–3_ [Equation (26)]:

(35) $${\text{dH}}_{23}^{\text{c}}=\text{H}({\text{c}}_{3}+{\text{dc}}_{3},\text{V})-\{\text{H}({\text{c}}_{3}^{\text{s}},\text{dv})+\text{H}({\text{c}}_{3},\text{V}-\text{dv})\}$$

Again, by virtue of H(c_3_,V) = h_v_(c_3_)V (Equation (153) in “Appendix 4: Basic equations”):

(36) $$\begin{array}{l} \text{H}({\text{c}}_{3}+{\text{dc}}_{3},\text{V})={\text{h}}_{\text{v}}({\text{c}}_{3}+{\text{dc}}_{3})\text{V}\\ \text{H}({\text{c}}_{3}^{\text{s}},\text{V})={\text{h}}_{\text{v}}({\text{c}}_{3}^{\text{s}})\text{V}\\ \text{H}({\text{c}}_{3},\text{V}-\text{dv})={\text{h}}_{\text{v}}({\text{c}}_{3}^{\text{s}})(\text{V}-\text{dv})={\text{h}}_{\text{v}}({\text{c}}_{3}^{\text{s}})\text{V}-{\text{h}}_{\text{v}}({\text{c}}_{3}^{\text{s}})\text{dv} \end{array}$$

The heat involved in the process 2–3, dQ^c^_2–3_, is calculated by using (35) and (36) in (34):

(37) $${\text{dQ}}_{2-3}^{\text{c}}={\text{dH}}_{\text{v}}({\text{c}}_{3})\text{V}+\left[{\text{h}}_{\text{v}}({\text{c}}_{3})-{\text{h}}_{\text{v}}({\text{c}}_{3}^{\text{s}})\right]\text{dv}$$

where:

(38) $${\text{dh}}_{\text{v}}({\text{c}}_{3})={\text{h}}_{\text{v}}({\text{c}}_{3}+{\text{dc}}_{3})-{\text{h}}_{\text{v}}({\text{c}}_{3})$$

Now, we can apply the First Principle of Thermodynamics [Equation (9)] to the complete concentration process:

(39) $${\text{dH}}^{\text{c}}={\text{dW}}_{\text{inj}}^{\text{c}}+{\text{dQ}}^{\text{c}}$$

where the work involved is the work of injection. In this equation, dQ^c^ represents the heat measured by the isothermal titration calorimeter in the experiment of concentration. From Figure 3 and the values of dH^c^_1–2_ and dH^c^_2–3_ calculated with respectively Equations (28) and (34), we obtain:

(40) $$\begin{array}{ll} {\text{dH}}^{\text{c}} & ={\text{dH}}_{1-2}^{\text{c}}+{\text{dH}}_{2-3}^{\text{c}}\\ & ={\text{dW}}_{1-2}^{\text{c}}+{\text{dQ}}_{1-2}^{\text{c}}+{\text{dQ}}_{2-3}^{\text{c}} \end{array}$$

Combining Equations (39) and (40) yields:

(41) $${\text{dW}}_{\text{inj}}^{\text{c}}={\text{dW}}_{1-2}^{\text{c}}$$

(42) $${\text{dQ}}^{\text{c}}={\text{dQ}}_{1-2}^{\text{c}}+{\text{dQ}}_{2-3}^{\text{c}}$$

Note that according to (41), dW^c^_1–2_ is the work of injection in the process of concentration; because dQ^c^_1–2_ = -dW^c^_1–2_ (Equation (32)), dQ^c^_1–2_ can be considered the “injection heat”. We name this heat dQ^c^_inj_; then (42) can take the following form:

(43) $${\text{dQ}}^{\text{c}}={\text{dQ}}_{\text{inj}}^{\text{c}}+{\text{dQ}}_{2-3}^{\text{c}}$$

Now, it is possible to obtain the heat involved in the infinitesimal process of concentration, dQ^c^, inserting the value of dQ^c^_2–3_ (Equation (37) ) into (43):

(44) $${\text{dQ}}^{\text{c}}={\text{dQ}}_{\text{inj}}^{\text{c}}+{\text{dh}}_{\text{v}}({\text{c}}_{3})\text{V}+\left[{\text{h}}_{\text{v}}({\text{c}}_{3})-{\text{h}}_{\text{v}}({\text{c}}_{3}^{\text{s}})\right]\text{dv}$$

#### 3.3.2. Dilution experiment in 2-component systems

In this experiment we will consider similar states as those in the concentration process; because it is a dilution experiment, however, the change in composition from c_3_ to c_3_ + dc_3_ is produced by a titration with the solvent located in the syringe. The states in the titration process are:

(45) $$\begin{array}{l} {\text{H}}_{1}^{\text{d}}=\text{H}({\text{c}}_{3},\text{V})+\text{H}(0,\text{dv})\\ {\text{H}}_{2}^{\text{d}}=[\text{H}({\text{c}}_{3},\text{V}-\text{dv})+\text{H}(0,\text{dv})]+\text{H}({\text{c}}_{3},\text{dv})\\ {\text{H}}_{3}^{\text{d}}=\text{H}({\text{c}}_{3}+{\text{dc}}_{3},\text{V})+\text{H}({\text{c}}_{3},\text{dv}) \end{array}$$

The variation in enthalpy for the total process of titration is:

(46) $${\text{dH}}^{\text{d}}={\text{H}}_{3}^{\text{d}}-{\text{H}}_{1}^{\text{d}}$$

As in the concentration experiment presented in Figure 3, for the dilution experiment we consider similar processes 1–2 and 2–3 defined as:

(47) $${\text{dH}}_{1-2}^{\text{d}}={\text{H}}_{2}^{\text{d}}-{\text{H}}_{1}^{\text{d}}$$

(48) $${\text{dH}}_{2-3}^{\text{d}}={\text{H}}_{3}^{\text{d}}-{\text{H}}_{2}^{\text{d}}$$

and then:

(49) $${\text{dH}}^{\text{d}}={\text{dH}}_{1-2}^{\text{d}}+{\text{dH}}_{2-3}^{\text{d}}$$

The First Principle of Thermodynamics [Equation (9)] for the process 1–2 allows to write:

(50) $${\text{dH}}_{1-2}^{\text{d}}={\text{dW}}_{1-2}^{\text{d}}+{\text{dQ}}_{1-2}^{\text{d}}$$

The value of dH^d^_1–2_ is obtained by substituting the values of H^d^_1_ and H^d^_2_ (Equation (45)) into the definition of dH^d^_1–2_ (Equation (49)) and considering the property H(c_2_,V)=h_v_(c_2_)V (Equation (153) in “Appendix 4: Basic equations”):

(51) $${\text{dH}}_{1-2}^{\text{d}}=0$$

With this result, according to the First Principle of Thermodynamics [Equation (50)] for the process 1–2, yields:

(52) $${\text{dQ}}_{1-2}^{\text{d}}=-{\text{dW}}_{1-2}^{\text{d}}$$

For process 2–3, in which only a homogenizing process occurs, the work is zero and the First Principle of Thermodynamics [Equation (9)] for this process takes the form:

(53) $${\text{dH}}_{2-3}^{\text{d}}={\text{dQ}}_{2-3}^{\text{d}}$$

From this equation it is possible to calculate the value of dQ^d^_2–3_ by substituting the values H^d^_2_ and H^d^_3_ (Equation (45)) into the definition of dH^d^_2–3_ [Equation (48)] and considering the property H(c_2_,V)=h_v_(c_2_)V:

(54) $${\text{dQ}}_{2-3}^{\text{d}}={\text{dh}}_{\text{v}}({\text{c}}_{2})\text{V}+[{\text{h}}_{\text{v}}({\text{c}}_{2})-{\text{h}}_{1}\rho_{1}]\text{dv}$$

where:

(55) $${\text{dh}}_{\text{v}}({\text{c}}_{2})={\text{h}}_{\text{v}}({\text{c}}_{2}+{\text{dc}}_{2})-{\text{h}}_{\text{v}}({\text{c}}_{2})$$

(56) $${\text{h}}_{1}\rho_{1}={\text{h}}_{\text{v}}(0)$$

and h_1_ and ρ_1_ are the enthalpy and the density, respectively, of component 1 in the pure state. Now, the First Principle of Thermodynamics (Equation (9)) for the complete titration process of dilution gives:

(57) $${\text{dH}}^{\text{d}}={\text{dW}}_{\text{inj}}^{\text{d}}+{\text{dQ}}^{\text{d}}$$

where dW^d^_inj_ is the work employed in the process of titration and dQ^d^ is the heat measured by the isothermal calorimeter in the experiment of dilution. Equation (49) expresses dH^d^ as the sum of the two contributions dH^d^_1–2_ and dH^d^_2–3_. With the First Principle of Thermodynamics applied to the process 1–2 [Equation (50)] and to the process 2–3 [Equation (53)], we have:

(58) $${\text{dH}}^{\text{d}}=({\text{dW}}_{1-2}^{\text{d}}+{\text{dQ}}_{1-2}^{\text{d}})+{\text{dQ}}_{2-3}^{\text{d}}$$

Putting (57) and (58) equal and reorganizing yields:

(59) $${\text{dW}}_{\text{inj}}^{\text{d}}={\text{dW}}_{1-2}^{\text{d}}$$

(60) $${\text{dQ}}^{\text{d}}={\text{dQ}}_{\text{inj}}^{\text{d}}+{\text{dQ}}_{2-3}^{\text{d}}$$

with dQ^d^_inj_ = dQ^d^_1–2_= -dWd_1–2_. Then substituting the value of dQd_2–3_ expressed by Equation (54) into (60) we obtain:

(61) $${\text{dQ}}^{\text{d}}={\text{dQ}}_{\text{inj}}^{\text{d}}+{\text{dh}}_{\text{v}}({\text{c}}_{2})\text{V}+[{\text{h}}_{\text{v}}({\text{c}}_{2})-{\text{h}}_{1}\rho_{1}]\text{dv}$$

#### 3.3.3. Concentration-dilution experiment in 3-component systems

In this experiment, a solution of component 2 in a solvent (component 1) is titrated with a stock solution of component 3 in the same solvent. For State 1 as in Figure 3, we consider that the solution in the interior of the vessel is composed of components 2 and 3 in component 1 with the concentrations c_2_ = n_2_/V and c_3_ = n_3_/V, respectively. We consider that the volume dv, before it is introduced into the vessel, has a concentration c^s^_3_. For convenience, we consider the 3-component system as fractionalized, being composed of component 1 and a fraction F containing components 2 and 3. The composition of the fraction F will be expressed as a function of the variables c_F_ and x_f3_, as defined by Equations (18) and (19). Thus, the enthalpy H_1_ of State 1 is:

(62) $$\begin{array}{ll} {\text{H}}_{1} & =\text{H}({\text{c}}_{\text{F}},{\text{x}}_{\text{f}3},\text{V})+\text{H}({\text{c}}_{3}^{\text{s}},\text{dv})\\ & ={\text{h}}_{\text{v}}({\text{c}}_{\text{F}},{\text{x}}_{\text{f}3})\text{V}+{\text{h}}_{\text{v}}({\text{c}}_{3}^{\text{s}}\text{dv}) \end{array}$$

In State 2, while a volume dv of stock solution with a concentration c^s^_3_ is titrated, an equal volume dv of solution with the composition c_F_ and x_f3_, is pushed out from the vessel. The enthalpy of this state is:

(63) $$\begin{array}{ll} {\text{H}}_{2} & =\left[\text{H}({\text{c}}_{\text{F}},{\text{x}}_{\text{f}3},\text{V}-\text{dv})+\text{H}({\text{c}}_{3}^{\text{s}},\text{dv})\right]+\text{H}({\text{c}}_{\text{F}},{\text{x}}_{\text{f}3},\text{dv})\\ & ={\text{h}}_{\text{v}}({\text{c}}_{\text{F}},{\text{x}}_{\text{f}3})\text{V}+{\text{h}}_{\text{v}}({\text{c}}_{3}^{\text{s}})\text{dv} \end{array}$$

After homogenization, we have a volume V with composition c_F_ + dc_F_ and x_f3_ + dx_f3_ and a volume dv in the drainage capillary with the composition c_F_ and x_f3_. In this way, the enthalpy of State 3 is:

(64) $$\begin{array}{ll} {\text{H}}_{3} & =\left[\text{H}({\text{c}}_{\text{F}}+{\text{dc}}_{\text{F}},{\text{x}}_{\text{f}3}+{\text{dx}}_{\text{f}3},\text{V})+\text{H}({\text{c}}_{\text{F}},{\text{x}}_{\text{f}3},\text{dv})\right]\\ & ={\text{h}}_{\text{v}}({\text{c}}_{\text{F}}+{\text{dc}}_{\text{F}},{\text{x}}_{\text{f}3}+{\text{dx}}_{\text{f}3})\text{V}+{\text{h}}_{\text{v}}({\text{c}}_{\text{F}},{\text{x}}_{\text{f}3})\text{dv} \end{array}$$

Applying the First Principle of Thermodynamics (Equation (9)) to this experiment gives:

(65) $$\text{dH}={\text{dW}}_{\text{inj}}+{\text{dQ}}_{\text{inj}}$$

Considering the processes 1–2 and 2–3 as in the above experiments, we arrive at the following equations:

(66) $${\text{dW}}_{\text{inj}}=-{\text{dQ}}_{\text{inj}}=-{\text{dQ}}_{1-2}$$

(67) $$\text{dQ}={\text{dQ}}_{\text{inj}}+{\text{dh}}_{\text{v}}({\text{c}}_{\text{F}},{\text{x}}_{\text{f}3})\text{V}+\left[{\text{h}}_{\text{v}}({\text{c}}_{\text{F}},{\text{x}}_{\text{f}3})-{\text{h}}_{\text{v}}({\text{c}}_{3}^{\text{s}})\right]\text{dv}$$

### 3.4. Heats of interaction between 2 components in the high dilution region

Next, we will discuss the protocol for measuring the heat of interaction between two components in solution in the high dilution region (see “Appendix 3: The region of high dilution”). We assume that titration proceeds as an infinitesimal process.

The first experiment is the titration of a solution of component 2 with a stock solution of component 3. Initially, the concentration of component 2 in the vessel is c_2_, and the concentration of component 3 in the stock solution is c^s^_3_, with dv being the volume of titration. The solvent in the two solutions is the same. The heat measured in this experiment is named dQ^(3)^ where the superindex (3) indicates that a 3-component system is considered. The second experiment is a concentration experiment, in which the solvent is titrated with a volume dv of a stock solution of component 3. As in the first experiment the titrated volume of the stock solution of concentration c^s^_3_ is dv. In this case, the heat measured is dQ^(2)c^ where the superindex (2) indicates that a 2-component system is considered. The third experiment is a dilution experiment, in which a solution of component 2 is titrated with the solvent. Initially, the concentration of component 2 in the solvent is c_2_. The heat measured in this case is dQ(2)d. The fourth experiment is the tritration of the solvent with itself. In this experiment the heat measured is dQ_inj_(1) where the superindex (1) indicates that a 1-component system is considered in this experiment. We will define the following amounts:

(68) $$\begin{array}{c} {\text{dq}}^{\left(3\right)}={\text{dQ}}^{\left(3\right)}-{\text{dQ}}_{\text{inj}}^{\left(3\right)}\\ {\text{dq}}^{(2)\text{c}}={\text{dQ}}^{(2)\text{c}}-{\text{dQ}}_{\text{inj}}^{(2)\text{c}}\\ {\text{dq}}^{(2)\text{d}}={\text{dQ}}^{(2)\text{d}}-{\text{dQ}}_{\text{inj}}^{(2)\text{d}} \end{array}$$

where dQ^(3)^_inj_, dQ(2)c_inj_, dQ(2)d_inj_ are the heats of titration in the three firsts experiments. We suppose that the heats of injection can be estimated by the titration of component 1 with itself (fourth experiment), dQ^(1)^_inj_:

(69) $${\text{dQ}}_{\text{inj}}^{\left(1\right)}\approx {\text{dQ}}^{(2)\text{d}}\approx {\text{dQ}}_{\text{inj}}^{(2)\text{c}}\approx {\text{dQ}}_{\text{inj}}^{\left(3\right)}$$

The heat, dq_3;1,2_, measured from the protocol with component 3 as the titrant is defined as:

(70) $${\text{dq}}_{3;1,2}={\text{dq}}^{\left(3\right)}-\left\{{\text{dq}}^{(2)\text{c}}+{\text{dq}}^{(2)\text{d}}\right\}$$

The notation “dq_3;1,2_” means that a solution of components 1 and 2 is titrated with a stock solution of component 3. By substituting the values of dQ^(3)^ (Equation (67)), dQ^(2)c^ (Equation (44)) and dQ^(2)d^ [Equation (61)], we arrive at:

(71) $$\begin{array}{l} {\text{dq}}^{\left(3\right)}={\text{dh}}_{\text{v}}({\text{c}}_{\text{F}},{\text{x}}_{\text{f}3})\text{V}+\left[{\text{h}}_{\text{v}}({\text{c}}_{\text{F}},{\text{x}}_{\text{f}3})-{\text{h}}_{\text{v}}({\text{c}}_{3}^{\text{s}})\right]\text{dv}\\ {\text{dq}}^{(2)\text{c}}={\text{dh}}_{\text{v}}({\text{c}}_{3})\text{V}+\left[{\text{h}}_{\text{v}}({\text{c}}_{3})-{\text{h}}_{\text{v}}({\text{c}}_{3}^{\text{s}})\right]\text{dv}\\ {\text{dq}}^{(2)\text{d}}={\text{dh}}_{\text{v}}({\text{c}}_{2})\text{V}+[{\text{h}}_{\text{v}}({\text{c}}_{2})-{\text{h}}_{1}\rho_{1}]\text{dv} \end{array}$$

Combining Equations (71) and (70) yields:

(72) $${\text{dq}}_{3;1,2}=\text{V}\;[{\text{dh}}_{\text{v}}({\text{c}}_{\text{F}},{\text{x}}_{\text{f}3})-{\text{dh}}_{\text{v}}({\text{c}}_{3})-{\text{dh}}_{\text{v}}({\text{c}}_{2})]+[{\text{h}}_{\text{v}}({\text{c}}_{\text{F}},{\text{x}}_{\text{f}3})-{\text{h}}_{\text{v}}({\text{c}}_{3})-{\text{h}}_{\text{v}}({\text{c}}_{2})+{\text{h}}_{1}\rho_{1}]\text{dv}$$

For convenience, we define f_v_ as:

(73) $${\text{f}}_{\text{v}}({\text{c}}_{\text{F}},{\text{x}}_{\text{f}3})=[{\text{h}}_{\text{v}}({\text{c}}_{\text{F}},{\text{x}}_{\text{f}3})-{\text{h}}_{\text{v}}({\text{c}}_{3})-{\text{h}}_{\text{v}}({\text{c}}_{2})+{\text{h}}_{1}\rho_{1}]$$

where c_2_ and c_3_ can be written as functions of c_F_ and x_f3_ as c_2_ = (1-x_f3_) c_F_ and c_2_ = x_f3_ c_F_.

We are interested in the following amount:

(74) $$\frac{{\text{dq}}_{3;1,2}}{\text{dv}}\equiv \left\{\begin{array}{c} \text{Heat}\;\text{obtained}\;\text{from}\;\text{the}\;\text{protocol}\;\text{per}\;\text{unit}\\ \text{of}\;\text{volume}\;\text{of}\;\text{titrant}\;\text{solution}\;\text{when}\\ \text{component}\;3\;\text{is}\;\text{the}\;\text{titrant}\;\text{component}\;\text{and}\\ \text{the}\;\text{volume}\;\text{of}\;\text{titration}\;\text{is}\;\text{infinitesimal} \end{array}\right\}$$

Substituting (72) and (73) into (74) yields:

(75) $$\frac{{\text{dq}}_{3;1,2}}{\text{dv}}=\text{V}\frac{{\text{df}}_{\text{v}}}{\text{dv}}+{\text{f}}_{\text{v}}$$

Now, we assume that the solutions in the cell are diluted solutions. In general, a molar property depends on x_F_ (amount of fraction F) and x_f3_ (composition of F). In previous works [4–6] we have shown that a solution is diluted when its molar properties can be approximated by first order Taylor’s expansions for x_F_ close to zero. The region of concentrations for which this approximation holds is a high dilution region. Function f_v_ in Equation (73) is expressed in terms of h_v_(c_F_,x_f3_), h_v_(c_3_), h_v_(c_2_) and h_1_ρ_1_. From Equations (143) or (153) (in “Appendix 4: Basic equations”), h_v_ is a “volumetric enthalpy” since h_v_ = H/V, H being the total enthalpy of the system and V the total volume of the system. consequently, h_v_ is expressed in “units of enthalpy per unit of volume”. Furthermore, h_v_ = h/v, where h is the molar enthalpy and v the molar volume, we can thus consider dilute solutions in f_v_ by using the first order Taylor’s expansions of molar volumes and molar enthalpies. The details of our calculations are presented in “Appendix 4: Basic equations”. By substituting the expressions of h_v_(x_F_,x_f3_), h_v_(c_2_) and h_v_(c_3_) for their dilute solutions [Equations (152) and (155)] in (73), we obtain that:

(76) $${\text{f}}_{\text{v}}({\text{c}}_{\text{F}},{\text{x}}_{\text{f}3})={\text{c}}_{\text{F}}\left[{\text{h}}_{\text{F};1}^{\text{o}}-{\text{h}}_{2;1}^{\text{o}}{\text{x}}_{\text{f}2}-{\text{h}}_{3;1}^{\text{o}}{\text{x}}_{\text{f}3}\right]-\rho_{1}{\text{h}}_{1}{\text{c}}_{\text{F}}\left[{\text{v}}_{\text{F},1}^{\text{o}}-{\text{v}}_{2,1}^{\text{o}}{\text{x}}_{\text{f}2}-{\text{v}}_{3,1}^{\text{o}}{\text{x}}_{\text{f}3}\right]$$

As indicated in “Appendix 2: Limits at infinite dilution in multicomponent systems,” the partial molar volume and the partial molar enthalpy of fraction F can be broken down into two parts. The first is the contribution of (non interacting) components of fraction F:

(77) $$\begin{array}{c} {\text{h}}_{\text{F},1}^{\emptyset }({\text{X}}_{\text{f}3})={\text{h}}_{2,1}^{\text{o}}{\text{X}}_{\text{f}2}+{\text{h}}_{3,1}^{\text{o}}{\text{X}}_{\text{f}3}\\ {\text{v}}_{\text{F},1}^{\emptyset }({\text{X}}_{\text{f}3})={\text{v}}_{2,1}^{\text{o}}{\text{X}}_{\text{f}2}+{\text{v}}_{3,1}^{\text{o}}{\text{X}}_{\text{f}3} \end{array}$$

The second is the contribution from the interactions between components of the fraction:

(78) $$\begin{array}{l} \Delta {\text{h}}_{\text{F},1}^{\text{o}}({\text{X}}_{\text{f}3})={\text{h}}_{\text{F},1}^{\text{o}}({\text{X}}_{\text{f}3})-{\text{h}}_{\text{F},1}^{\emptyset }({\text{X}}_{\text{f}3})\\ \Delta {\text{v}}_{\text{F},1}^{\text{o}}({\text{X}}_{\text{f}3})={\text{v}}_{\text{F},1}^{\text{o}}({\text{X}}_{\text{f}3})-{\text{v}}_{\text{F},1}^{\emptyset }({\text{X}}_{\text{f}3}) \end{array}$$

Using (77) and (78), Equation (76) takes the form:

(79) $${\text{f}}_{\text{v}}({\text{c}}_{\text{F}},{\text{X}}_{\text{f}3})={\text{c}}_{\text{F}}\Delta {\text{h}}_{\text{F},1}^{\text{o}}({\text{X}}_{\text{f}3})-\rho_{1}{\text{h}}_{1}{\text{c}}_{\text{F}}\Delta {\text{v}}_{\text{F},1}^{\text{o}}({\text{X}}_{\text{f}3})$$

Therefore, if the solutions are sufficiently diluted, the function f_v_ shows the contribution of the interaction enthalpy and the interaction volume of the fraction F. The differential can be expressed as:

(80) $$\frac{{\text{df}}_{\text{v}}}{\text{dv}}={\left(\frac{\partial {\text{f}}_{\text{v}}}{\partial {\text{c}}_{\text{F}}}\right)}_{{\text{X}}_{\text{f}3}}\frac{{\text{dc}}_{\text{F}}}{\text{dv}}+{\left(\frac{\partial {\text{f}}_{\text{v}}}{\partial {\text{X}}_{\text{f}3}}\right)}_{{\text{c}}_{\text{F}}}\frac{{\text{dx}}_{\text{f}3}}{\text{dv}}$$

By combining the equation for df_v_/dv [Equation (80)], f_v_ (Equation (79)) and those for c_F_ = c_F_(v) and t_f3_ = t_f3_(v) given in (20) and (21), we obtain:

(81) $$\frac{{\text{dq}}_{3;1,2}}{\text{dv}}={\text{c}}_{3}^{\text{s}}\left[\Delta {\text{h}}_{\text{F};1}^{\text{o}}+\frac{\text{d}\Delta {\text{h}}_{\text{F}:1}^{\text{o}}}{{\text{dx}}_{\text{f}3}}(1-{\text{X}}_{\text{f}3})\right]-\rho_{1}{\text{h}}_{1}{\text{c}}_{3}^{\text{s}}\left[\Delta {\text{v}}_{\text{F};1}^{\text{o}}+\frac{\text{d}\Delta {\text{v}}_{\text{F};1}^{o}}{{\text{dx}}_{\text{f}3}}(1-{\text{X}}_{\text{f}3})\right]$$

In the definition of dq_3;1,2_/dv [Equation (74)] the volume of tritration is considered to be infinitesimally small. Thus, in the calculations for dc_F_/dv and dt_f3_/dv in Equation (81), we assume that exp(-v/V) ≈ 1. From the Equation (125) (see “Appendix 2: Limits at infinite dilution in multicomponent systems”) it is possible to write:

(82) $$\begin{array}{l} \Delta {\text{h}}_{3;1,2}^{\Delta }={\Delta \text{h}}_{\text{F};1}^{\text{o}}+\frac{\text{d}\Delta {\text{h}}_{\text{F};1}^{\text{o}}}{{\text{dx}}_{\text{f}3}}(1-{\text{X}}_{\text{f}3})\\ \Delta {\text{v}}_{3;1,2}^{\Delta }=\Delta {\text{v}}_{\text{F};1}^{\text{o}}+\frac{\text{d}\Delta {\text{v}}_{\text{F};1}^{\text{o}}}{{\text{dX}}_{\text{f}3}}(1-{\text{x}}_{\text{f}3}) \end{array}$$

Now, (81) takes the form:

(83) $$\frac{{\text{dq}}_{3;2,1}}{\text{dv}}={\text{c}}_{3}^{\text{s}}\Delta {\text{h}}_{3;2,1}^{\Delta }-\rho_{1}{\text{h}}_{1}{\text{c}}_{3}^{\text{s}}\Delta {\text{v}}_{3;2,1}^{\Delta }$$

Since dn^s^_3_=cs_3_ dv, then:

(84) $$\frac{{\text{dq}}_{3;1,2}}{\text{dv}}={\text{c}}_{3}^{\text{s}}\frac{{\text{dq}}_{3;1,2}}{{\text{dn}}_{3}^{\text{s}}}$$

where:

(85) $$\frac{{\text{dq}}_{3;1,2}}{{\text{dn}}_{3}^{\text{s}}}\equiv \left\{\begin{array}{c} \text{Heat}\;\text{obtained}\;\text{from}\;\text{the}\;\text{protocol}\;\\ \text{per}\;\text{mol}\;\text{of}\;\text{titrant}\;\text{component}\;\text{when}\\ \text{component}\;3\;\text{is}\;\text{the}\;\text{titrant}\;\text{component}\;\text{and}\\ \text{when}\;\text{the}\;\text{titration}\;\text{amount}\;\text{is}\;\text{infinitesimal} \end{array}\right\}$$

By combining Equations (83) and (84), we obtain:

(86) $$\frac{{\text{dq}}_{3;1,2}}{{\text{dn}}_{3}^{\text{s}}}=\Delta {\text{h}}_{3;1,2}^{\Delta }-\rho_{1}{\text{h}}_{1}\Delta {\text{v}}_{3;1,2}^{\Delta }$$

## 4. Discussion

As it has been stated previously, a measured heat is obtained experimentally when a liquid is titrated with itself. Figure 2 shows the measurement of this heat when water is titrated with water at 30 °C. This result agrees with those that have been obtained by other authors [2]. This heat has been named as “blank machine” [2] or “instrumental heat” and its origin could be attributed to a possible difference in temperatures between the titrated volume and the cell. In the case of Figure 2, the room temperature was 20 °C and the temperature cell was 30 °C. That is, if a difference in temperature existed, the initial temperature T_i_ of the titrant volume would be less than the final temperature T_f_. According to equation:

(87) $${\text{Q}}_{\text{inj}}=\text{m}\times {\text{c}}_{\text{p}}\times \Delta \text{T}$$

where Q_inj_ is the heat obtained from the injection, m the mass of the titrant volume, c_p_ the specific heat capacity and ΔT = T_f_ - T_i_ we would expect a heat positive. The heat shown on Figure 2 is negative and therefore it is not possible to explain the heat observed on Figure 2 in terms of a “blank machine” or an “instrumental heat”. The merit of the equation ΔH = W_inj_ + Q [Equation (9)] is that it allows to take into account a heat measured by the calorimeter when a liquid is titrated with itself and the sign of this heat. Because it is necessary to apply work to the system in order to introduce an amount of liquid into the cell and push out an equivalent amount of liquid, this work must be positive. Since in this case Q_inj_ = -W_inj_, the heat measured must to be negative. The heat shown in Figures 2-II agrees with this prediction.

Contributions to Q_inj_ can be several as for example the friction between liquids (relative viscosities) and the friction between the liquid and the narrow bore tube of the needle. Recently [8] the following equation has been proposed that gives the temperature rise in a fluid from frictional flow in a tube:

(88) $$\Delta \text{T}=21\times 10^{-10}\frac{\mu \text{l}{\text{v}}^{\prime }}{\pi \rho {\text{c}}_{\text{p}}{\text{d}}^{4}}$$

where ΔT is the difference in temperature in K, μ is the fluid viscosity in centipoises, l is the length of the tube in cm, v’ is the volumetric flow rate in cm^3^ min^−1^, ρ is the fluid density in g cm^3^, C is the fluid heat capacity in J g^−1^ K^−1^, and d is the tube diameter in cm. For water flowing through a 0.4 mm diameter tube 30 cm long at 1 cm^3^ min^−1^, ΔT = 0.002 K. As it is stated by Equation (88) ΔT depends on the nature of the fluid through its viscosity, density and heat capacity, on the geometry of the calorimetric system through the diameter and length of the needle and to the conditions of the experiment through the volume flow rate. When combining Equations (87) and (88) it results an expected influence of the volumetric flow rate (v’) in Q_inj_. This fact was shown experimentally in the Figure 2.7 of ref. [2].

**Figure 5.**
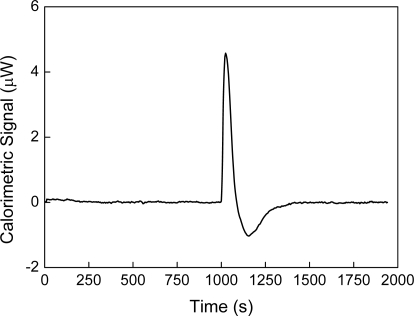
Calorimetric signal of the titration of toluene with toluene at 30 °C. The volume of titration was 200 μL.

Figure 5 shows the calorimetric signal of the titration of toluene with toluene. Unlike in Figure 1 in which all peaks are exothermic, in this case a minimum with a negative value (endothermic peak) was recorded. Usually, the syringe is at the temperature of the room, and the cell is at the fixed temperature of the experiment. This endothermic peak can be explained by the large volume of titration (which is 15% of the volume of the cell) and the difference in temperature between the cell and the room.

Therefore we can state that a characteristic of isothermal titration calorimetry is the necessity of very small volume according to two considerations: first, with a large volume, the temperature of the experiment is not kept constant, second, the validity of Equation (81) imposes very small titration volumes in order to assume that the heat obtained following the experimental the protocol is related to a partial molar enthalpy of interaction at infinite dilution and to a term proportional to a partial molar volume of interaction also at infinite dilution.

**Figure 6.**
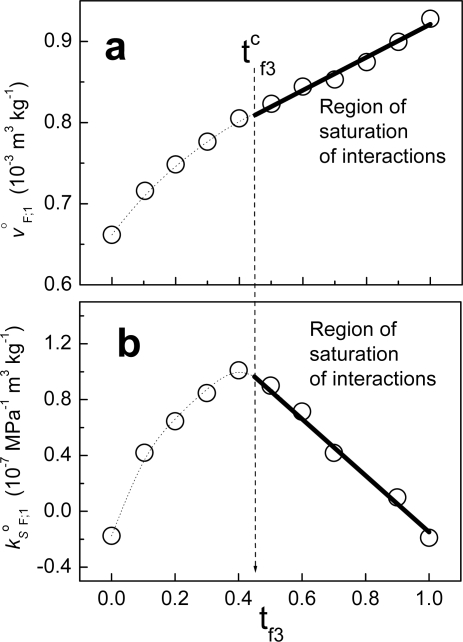
Specific partial volume at infinite dilution a) and specific partial adiabatic compressibility coefficient b) at infinite dilution in water at 30 °C, of a fraction F composed of non-charged polymeric particles (component 2) and decyltrimethyl-ammnonium bromide (component 3) as function of the mass fraction of component 3 in the fraction F. The solid line represents the region in which the interactions are saturated (data taken from ref. [5]).

In Equation (86), we have two contributions to the heat obtained from the given protocol. One is the partial molar enthalpy of interaction of component 3 within the limit of infinite dilution (Δh^Δ^_3;1,2_). The second contribution is – ρ_1_h_1_Δv^Δ^_3;1,2_. This term represents the enthalpy of a volume of solvent Δv^Δ^_3;1,2_ as a consequence of the protocol employed. In addition to this, it is possible to demonstrate that when the interactions between two components are maximum, the heat dq_3;1,2_/dns_3_ obtained is zero. In a previous work [5], we demonstrated that if the plot of j^o^_F;1_ as function of a variable of composition is linear for a range of compositions of F, then the interactions between the components of the fraction are maximum in that range. The composition variable employed was the mass fraction of component 3 in the fraction (t_f3_). Figure 6 shows an example when fraction F is composed of non-charged polymeric particles (component 2) and a cationic surfactant (component 3). The solvent in this case is water. From zero to t_f3_, the behavior is non-linear. Considering that the value tc_f3_ in units of molar fractions is x^c^_f3_, at this composition the partial property of F takes the value:

(89) $${\text{j}}_{\text{F};1}^{\text{o}}({\text{x}}_{\text{f}3}^{\text{c}})={\text{x}}_{\text{f}2}^{\text{c}}{\text{j}}_{2;1,3}^{\Delta }({\text{x}}_{\text{f}3}^{\text{c}})+{\text{x}}_{\text{f}3}^{\text{c}}{\text{j}}_{3;1,2}^{\Delta }({\text{x}}_{\text{f}3}^{\text{c}})$$

where x_f2_= 1-x_f3_. Above the value xc_f3_, j^o^_F;1_ can be written as:

(90) $${\text{j}}_{\text{F};1}^{\text{o}}={\text{X}}_{\text{f}2}{\text{j}}_{\text{F};1}^{\text{o}}({\text{x}}_{\text{f}3}^{\text{c}})+{\text{X}}_{\text{f}3}{\text{j}}_{3;1}^{\text{o}}$$

where:

(91) $${\text{X}}_{\text{f}3}=\frac{{\text{x}}_{\text{f}3}-{\text{x}}_{\text{f}3}^{\text{c}}}{{\text{x}}_{\text{f}2}^{\text{c}}}$$

and X_f2_= 1-X_f3_. When we write jΔ_2;1,3_ and j^Δ^_3;1,2_, we assume [4–6] that concomitantly component 2 is in the presence of components 1 and 3 and component 3 is in the presence of components 1 and 2. Thus the notation j^o^_F;1_ = x_f2_ j^Δ^_2;1,3_ + x_f3_ j^Δ^_3;1,2_ indicates that, F is composed of components 2 and 3, which are interacting in a medium (component 1). On the other hand, j^o^_2;1_ and j^o^_3;1_ indicate that component 2 is alone in component 1 and that component 3 is alone in component 1. Therefore, if we write j^o^_F;1_ = x_f2_ j^o^_2;1_ + x_f3_ j^o^_3;1_ we assume that fraction F is composed of components 2 and 3, which are not interacting.

This is the case for Equation (90), where fraction F is composed of a fraction of constant composition (with partial property j^o^_F;1_(xc_f3_)) and an amount of component 3 (with partial property j^o^_3;1_) and these components are not interacting. In other words [5,6], in a region of saturation of interactions, component 2 is interacting with a part of component 3 to form a fraction with constant composition. A fraction with constant composition is named a “pseudo-component [4–6].” This pseudo-component, composed of 2 and a part of 3, does not interact with the rest of component 3. A saturation of interactions is related to the formation of pseudo-components.

By substituting the equation for j^o^_F;1_ in the region of saturation (Equation (90)) in the equation for calculating j^o^_3;1,2_ from j^o^_F;1_ (Equation (117)) and bearing in mind that:

(92) $$\frac{{\text{dj}}_{\text{F};1}^{\text{o}}}{{\text{dx}}_{\text{f}3}}=\frac{{\text{dj}}_{\text{F};1}^{\text{o}}}{{\text{dX}}_{\text{f}3}}\frac{{\text{dX}}_{\text{f}3}}{{\text{dx}}_{\text{f}3}}$$

we obtain that:

(93) $$\Delta {\text{j}}_{3;2,1}^{\Delta }={\text{j}}_{3;2,1}^{\Delta }-{\text{j}}_{3;2,1}^{\text{o}}={\text{j}}_{3;1}^{\text{o}}-{\text{j}}_{3;1}^{\text{o}}=0$$

Substituting this result into Equation (86) we obtained that in the region of saturation of interactions:

(94) $$\frac{{\text{dq}}_{3;1,2}}{{\text{dn}}_{3}^{\text{s}}}=0$$

Another interesting problem in isothermal titration calorimetry is the following: is there a relationship between the experiments carried out when component 3 is the titrant and when component 2 is the titrant? We can answer this question as follows: the heat generated when component 3 is the titrant can be obtained from (86), dq_3;1,2_/dns_3_. In the same way, the heat obtained when component 2 is the titrant can be written as:

(95) $$\frac{{\text{dq}}_{2;1,3}}{{\text{dn}}_{2}^{\text{s}}}=\Delta {\text{h}}_{2;1,3}^{\Delta }-\rho_{1}{\text{h}}_{1}\Delta {\text{v}}_{2;1,3}^{\Delta }$$

Next we can derivate dq_3;1,2_/dns_3_ in Equation (86) with respect to x_f3_ and multiply by x_f2_, and we can also derivate dq_2;1,3_/dns_2_ with respect to x_f3_ and multiply by x_f3_. By adding the results and using Equation (123) (in Appendix 1: “Limits at infinite dilution in multicomponent systems”) for enthalpies and volumes:

(96) $${\text{x}}_{\text{f}2}\frac{\text{d}\Delta {\text{h}}_{2;1,3}^{\Delta }}{{\text{dx}}_{\text{f}3}}+{\text{x}}_{\text{f}3}\frac{\text{d}\Delta {\text{h}}_{3;1,2}^{\Delta }}{{\text{dx}}_{\text{f}3}}=0$$

(97) $${\text{x}}_{\text{f}2}\frac{\text{d}\Delta {\text{v}}_{2;1,3}^{\Delta }}{{\text{dx}}_{\text{f}3}}+{\text{x}}_{\text{f}3}\frac{{\text{dv}}_{3;1,2}^{\Delta }}{{\text{dx}}_{\text{f}3}}=0$$

we obtain:

(98) $${\text{x}}_{\text{f}2}\frac{\text{d}}{{\text{dx}}_{\text{f}3}}\left(\frac{{\text{dq}}_{2;1,3}}{{\text{dx}}_{\text{f}3}}\right)+{\text{x}}_{\text{f}3}\frac{\text{d}}{{\text{dx}}_{\text{f}3}}\left(\frac{{\text{dq}}_{3;1,2}}{{\text{dx}}_{\text{f}3}}\right)=0$$

This is an equation of the Gibbs-Duhem type that relates the heats of interaction obtained when components 2 and 3 are the titrant components.

**Figure 7.**
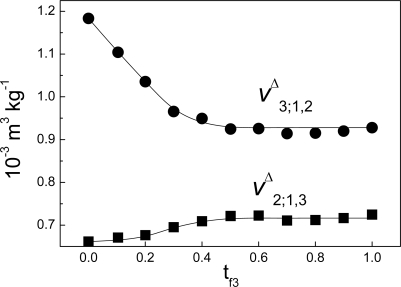
Partial volumes at infinite dilution of non-charged polymeric particles, v^Δ^_2;1,3_, and a cationic surfactant (C_10_-TAB), vΔ_3;1,2_, as function t_f3_ (data taken from ref. [5]).

**Figure 8.**
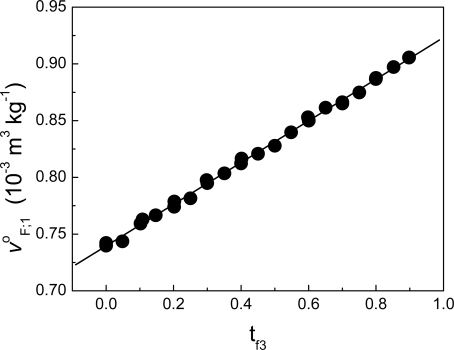
Specific partial volume at infinite dilution in water at 30 °C, of the fraction F composed of Lysozyme (component 2) and decyltrimethylammnonium bromide (component 3) as function of the mass fraction of component 3 in fraction F. Because the behavior of v^o^_F;1_ is very close to linear, the interaction term Δv^o^_F;1_ can be neglected (data taken from ref. [5]).

From equation ΔH = Q it is commonly assumed the heat measured by an ITC can be related to the variation of enthalpy; many papers and books in biochemistry and biophysics have reported results on this link. In this work, we have demonstrated that the equation ΔH = Q does not hold for isothermal titration calorimetry and that the true equation is ΔH = W_inj_ + Q, which involves a term of work. In addition, we have found that the heat obtained from the usual protocol employed in the determination of the heat of interaction dq_3;1,2_/dns_3_ between two components (Equation (86)) involves both a variation of enthalpy and a variation of volume. In general Δv^Δ^_3;1,2_ is not zero. As example of this, Figure 7 shows the case of the interaction between non-charged polymeric particles and a surfactant. On the other hand, if there were no link between the variation of enthalpy and the heat of interaction measured by ITC this would affect the results of heats of interaction obtained with the technique, particularly in biophysical applications. This paradox can be solved as follows: models have been proposed [9–11] that indicate that the variation in volume for protein unfolding is very small. In addition it has been found experimentally that the variation in volume during the denaturation of lysozyme by a strong denaturant is very close to zero [12]. In our case, we have found that Δv^Δ^_F;1_ can be neglected in the process of binding deciltrimethylammonium bromide to lysozyme [5] (see Figure 8). Supposing Δv^Δ^_F;1_ ≈ 0 in Equation (125) (in Appendix 1: “Limits at infinite dilution in multicomponent systems”), then:

(99) $$\Delta {\text{v}}_{3;1,2}^{\Delta }\approx 0$$

Considering that Equation (99) holds in general for a process involving protein unfolding, substituting this result into the equation of dq_3;1,2_/dns_3_ (Equation (86)) yields for this type of processes:

(100) $$\frac{{\text{dq}}_{3;1,2}}{{\text{dn}}_{3}^{\text{s}}}\approx \Delta {\text{h}}_{3;1,2}^{\Delta }$$

Another possibility is that for processes of biophysical interest, the approximation |ρ_1_h_1_Δv^Δ^_3;1,2_|≪ |Δh^Δ^_3;1,2_| holds.

## 5. Conclusions

In this work we have studied in detail the thermodynamics of the titration process in isothermal titration calorimeters with full cells. We have shown that the equation ΔH = Q does not hold for this type of calorimeters because it cannot explain the heat obtained when a liquid is titrated with itself. In its place, we propose the equation ΔH = W_inj_ + Q. The heat of interaction between two components is usually determined from a protocol composed of a number of simple titration experiments. Using the equation ΔH = W_inj_ + Q and the thermodynamic tools developed in our previous works for multicomponent systems at infinite dilution, we show that in an infinitesimal titration, the heat of interaction per mole of titrant component is related to the partial enthalpy of interaction at infinite dilution and to the partial volume of interaction of the titrant component also at infinite dilution. This information can be essential in order to link theoretical models to experimental measurements. Another interesting conclusion is that for this type of calorimeters the variation in enthalpy equals the variation in internal energy.

## Figures and Tables

**Table 1.** Isothermal titration calorimeters that are currently manufactured and the method employed by each (full cell or half-full cell).

**Calorimeter (Company)**	**Type of method: full cell or half-full cell**
iTC_200_ (Microcal Inc.)	Full Cell (1)
AUTO iTC_200_ (Microcal Inc.)	Full Cell (1)
VP-ITC (Microcal Inc.)	Full Cell (1)
Nano ITC 2G (TA Instruments)	Both, but the full cell method is most often used and is the strongly recommended method (2)
TAM 2277 (TA Instruments)	Both, but the half-full cell method is most often used and is the strongly recommended method (2)
TAM III ITC (TA Instruments)	Both, but the half-full cell method is most often used and is the strongly recommended method (2)

(1) Technical information supplied by MicroCal Inc.

(2) Technical information supplied by TA Instruments.

## Appendix 1. Fraction of a System and Fraction Variables

An extensive thermodynamic property J at constant temperature and pressure can be written in a “description by components” as:

(101) $$\text{J}=\text{J}({\text{n}}_{1},{\text{n}}_{2},{\text{n}}_{3})$$

where n_1_, n_2_ and n_3_ are the number of moles of the components 1, 2 and 3, respectively. The Gibbs Equation [13] for J takes the form:

(102) $$\text{dJ}={\text{j}}_{1;2,3}{\text{dn}}_{1}+{\text{j}}_{2;1,3}{\text{dn}}_{2}+{\text{j}}_{3;1,2}{\text{dn}}_{3}$$

where the partial properties j_1;2,3_, j_2;1,3_ and j_3;1,2_ are the partial properties of components 1, 2 and 3, respectively, defined as:

(103) $$\begin{array}{l} {\text{j}}_{1;2,3}({\text{x}}_{2},{\text{x}}_{3})={\left(\frac{\partial \text{J}({\text{n}}_{1},{\text{n}}_{2},{\text{n}}_{3})}{\partial {\text{n}}_{1}}\right)}_{{\text{n}}_{2},{\text{n}}_{3}}\\ {\text{j}}_{2;1,3}({\text{x}}_{2},{\text{x}}_{3})={\left(\frac{\partial \text{J}({\text{n}}_{1},{\text{n}}_{2},{\text{n}}_{3})}{\partial {\text{n}}_{2}}\right)}_{{\text{n}}_{1},{\text{n}}_{3}}\\ {\text{j}}_{3;1,2}({\text{x}}_{2},{\text{x}}_{3})={\left(\frac{\partial \text{J}({\text{n}}_{1},{\text{n}}_{2},{\text{n}}_{3})}{\partial {\text{n}}_{3}}\right)}_{{\text{n}}_{1},{\text{n}}_{2}} \end{array}$$

where x_2_ and x_3_ are the molar fraction of components 2 and 3, respectively. By notation, we understand that j_1;2,3_ means “the partial property of component 1 in the presence of components 2 and 3”. The notations j_2;1,3_ and j_3;1,2_ are interpreted in the same way.

A fraction of a system [4–6] is defined as a thermodynamic entity with an internal composition that groups several components. If we suppose a fraction F is composed of components 2 and 3, the property J can be written as a “description by fractions” as:

(104) $$\text{J}=\text{J}({\text{n}}_{1},{\text{n}}_{\text{F}},{\text{x}}_{\text{f}3})$$

where the new variables (fraction variables) are the total number of moles of the fraction F, n_F_ = n_2_ + n_3_, and x_f3_ = n_3_/(n_2_ + n_3_), which are related to the composition of F. The Gibbs equation for Equation (104) takes the form:

(105) $$\text{dJ}={\text{j}}_{1;\text{F}}{\text{dn}}_{1}+{\text{j}}_{\text{F};1}{\text{dn}}_{\text{F}}+{\left(\frac{\partial \text{J}}{\partial {\text{x}}_{\text{f}3}}\right)}_{{\text{n}}_{1},{\text{n}}_{\text{F}}}{\text{dx}}_{\text{f}3}$$

where j_1;F_ and j_F;1_ are respectively:

(106) $$\begin{array}{l} {\text{j}}_{1;\text{F}}({\text{x}}_{\text{F}},{\text{x}}_{\text{f}3})={\left(\frac{\partial \text{J}({\text{n}}_{1},{\text{n}}_{\text{F}},{\text{x}}_{\text{f}3})}{\partial {\text{n}}_{1}}\right)}_{{\text{n}}_{\text{F}},{\text{x}}_{\text{f}3}}\\ {\text{j}}_{\text{F};1}({\text{x}}_{\text{F}},{\text{x}}_{\text{f}3})={\left(\frac{\partial \text{J}({\text{n}}_{1},{\text{n}}_{\text{F}},{\text{x}}_{\text{f}3})}{\partial {\text{n}}_{\text{F}}}\right)}_{{\text{n}}_{1},{\text{x}}_{\text{f}3}} \end{array}$$

Again, by notation j_1;F_ means “the partial property of component 1 in the presence of fraction F” and in the same way, j_F;1_ means “the partial property of the fraction F in the presence of component 1”. By the technique of change of variable [14] we can write the partial properties j_1;F_ and j_F;1_ as function of the partial properties j_1;2,3_, j_2;1,3_, j_3;1,2_. The change of variable is:

(107) $$\begin{array}{l} {\text{n}}_{1}({\text{n}}_{1},{\text{n}}_{2},{\text{x}}_{\text{f}3})={\text{n}}_{1}\\ {\text{n}}_{2}({\text{n}}_{1},{\text{n}}_{\text{F}},{\text{x}}_{\text{f}3})=(1-{\text{x}}_{\text{f}3}){\text{n}}_{\text{F}}\\ {\text{n}}_{3}({\text{n}}_{1},{\text{n}}_{\text{F}},{\text{x}}_{\text{f}3})={\text{x}}_{\text{f}3}{\text{n}}_{\text{F}} \end{array}$$

By calculating the differentials of n_1_, n_2_ and n_3_ in (107), substituting dn_1_, dn_2_ and dn_3_ in (102), equaling the result to (105) and regrouping similar terms keeping in mind that n_1_, n_2_ and n_3_ are independent variables, we have:

(108) $${\text{j}}_{1;\text{F}}={\text{j}}_{1;2,3}$$

(109) $${\text{j}}_{\text{F};1}={\text{x}}_{\text{f}2}{\text{j}}_{2;1,3}+{\text{x}}_{\text{f}3}{\text{j}}_{3;1,2}$$

(110) $${\left(\frac{\partial \text{J}}{\partial {\text{x}}_{\text{f}3}}\right)}_{{\text{n}}_{1},{\text{n}}_{\text{F}}}={\text{n}}_{\text{F}}({\text{j}}_{3;1,2}-{\text{j}}_{2;1,3})$$

## Appendix 2. Limits at Infinite Dilution in Multicomponent Systems

The limit of j_F;1_ at infinite dilution is defined as:

(111) $${\text{lim}}_{\begin{array}{l} {\text{x}}_{\text{F}}\to 0\\ {\text{x}}_{\text{f}3\;\;\text{constant}} \end{array}}{\text{j}}_{\text{F};1}({\text{x}}_{\text{F}},{\text{x}}_{\text{f}3})={\text{j}}_{\text{F};1}(0,{\text{x}}_{\text{f}3})\equiv {\text{j}}_{\text{F};1}^{\text{o}}$$

The limit in (111) is taken when the concentration of the fraction tends to zero while its composition is kept constant. Under these conditions, the limits at infinite dilution of j_2;1,3_ and j_3;1,2_ are defined as:

(112) $${\text{lim}}_{\begin{array}{l} {\text{x}}_{\text{F}}\to 0\\ {\text{x}}_{\text{f}3\;\;\text{constant}} \end{array}}{\text{j}}_{2;1,3}({\text{x}}_{\text{F}},{\text{x}}_{\text{f}3})={\text{j}}_{2;1,3}(0,{\text{x}}_{\text{f}3})={\text{j}}_{2;1,3}^{\Delta }({\text{x}}_{\text{f}3})$$

(113) $${\text{lim}}_{\begin{array}{l} {\text{x}}_{\text{F}}\to 0\\ {\text{x}}_{\text{f}3\;\;\text{constant}} \end{array}}{\text{j}}_{3;1,2}({\text{x}}_{\text{F}},{\text{x}}_{\text{f}3})={\text{j}}_{3;1,2}(0,{\text{x}}_{\text{f}3})={\text{j}}_{3;1,2}^{\Delta }({\text{x}}_{\text{f}3})$$

Taking the limit at infinite dilution on both sides of (109) and substituting Equations (111)–(113) we obtain that:

(114) $${\text{j}}_{\text{F};1}^{\text{o}}={\text{x}}_{\text{f}2}{\text{j}}_{2;1,3}^{\Delta }+{\text{x}}_{\text{f}3}{\text{j}}_{3;1,2}^{\Delta }$$

In our previous work we showed that:

(115) $${\text{x}}_{\text{f}2}\frac{{\text{dj}}_{2;1,3}^{\Delta }}{{\text{dx}}_{\text{f}3}}+{\text{x}}_{\text{f}3}\frac{{\text{dj}}_{3;1,2}^{\Delta }}{{\text{dx}}_{\text{f}3}}=0$$

Derivating in (114) with respect to x_f3_ and combining the result with Equations (114) and (115) yields:

(116) $${\text{j}}_{2;1,3}^{\Delta }={\text{j}}_{\text{F};1}^{\text{o}}-{\text{x}}_{\text{f}3}\frac{{\text{dj}}_{\text{F};1}^{\text{o}}}{{\text{dx}}_{\text{f}3}}$$

(117) $${\text{j}}_{3;1,2}^{\Delta }={\text{j}}_{\text{F};1}^{\text{o}}+(1-{\text{x}}_{\text{f}3})\frac{{\text{dj}}_{\text{F};1}^{\text{o}}}{{\text{dx}}_{\text{f}3}}$$

The partial properties of 2 and 3 contribute due to their interaction. This effect can be measured as the effect on the partial property of a component due to the presence of the other component. In this way, we define the terms of interaction as:

(118) $$\Delta {\text{j}}_{2;1,3}^{\Delta }({\text{x}}_{\text{f}3})={\text{j}}_{2;1,3}^{\Delta }({\text{x}}_{\text{f}3})-{\text{j}}_{2;1}^{\text{o}}$$

(119) $$\Delta {\text{j}}_{3;1,2}^{\Delta }({\text{x}}_{\text{f}3})={\text{j}}_{3;1,2}^{\Delta }({\text{x}}_{\text{f}3})-{\text{j}}_{3;1}^{\text{o}}$$

Reorganizing (118) and (119) and substituting the values of j^Δ^_2;1,3_ and j^Δ^_3;1,2_ in (114) we have:

(120) $${\text{j}}_{\text{F};1}^{\text{o}}=\Delta {\text{j}}_{\text{F};1}^{\text{o}}+{\text{j}}_{\text{F};1}^{\emptyset }$$

where:

(121) $$\Delta {\text{j}}_{\text{F};1}^{\text{o}}={\text{x}}_{\text{f}2}\Delta {\text{j}}_{2;1,3}^{\Delta }+{\text{x}}_{\text{f}3}\Delta {\text{j}}_{3;1,2}^{\Delta }$$

and

(122) $${\text{J}}_{\text{F};1}^{\emptyset }={\text{x}}_{\text{f}2}{\text{j}}_{2;1}^{\text{o}}+{\text{x}}_{\text{f}3}{\text{j}}_{3;1}^{\text{o}}$$

Thus, from Equation (120) there are two contributions to the partial property of fraction F: j^∅︀^_F;1_, which does not consider the interaction between components 2 and 3, and Δj^o^_F;1_, which contains all contributions from the interaction between 2 and 3.

It is possible to see that terms of interaction also hold in a Gibbs-Duhem type equation. Reorganizing in (118) and (119) and substituting the values of j^Δ^_2;1,3_ and j^Δ^_3;12_ in the Gibbs-Duhem type equation for the partial properties (Equation (115)) we have:

(123) $${\text{x}}_{\text{f}2}\frac{\text{d}\Delta {\text{j}}_{2;1,3}^{\Delta }}{{\text{dx}}_{\text{f}3}}+{\text{x}}_{\text{f}3}\frac{\text{d}\Delta {\text{j}}_{3;1,2}^{\Delta }}{{\text{dx}}_{\text{f}3}}=0$$

As in the case of the partial properties, derivating (121) with respect to x_f3_ and combining with Equations (121) and (123) yields:

(124) $$\Delta {\text{j}}_{2;1,3}^{\Delta }=\Delta {\text{j}}_{\text{F};1}^{\text{o}}-{\text{x}}_{\text{f}3}\frac{\text{d}\Delta {\text{j}}_{\text{F};1}^{\text{o}}}{{\text{dx}}_{\text{f}3}}$$

(125) $$\Delta {\text{j}}_{3;1,2}^{\Delta }=\Delta {\text{j}}_{\text{F};1}^{\text{o}}+(1-{\text{x}}_{\text{f}3})\frac{\text{d}\Delta {\text{j}}_{\text{F};1}^{\text{o}}}{{\text{dx}}_{\text{f}3}}$$

## Appendix 3. The Region of High Dilution

Let a 3-component be. The Euler equation of the system in the description of fractions [Equation (104)] is:

(126) $$\text{J}={\text{n}}_{1}{\text{j}}_{1;\text{F}}+{\text{n}}_{\text{F}}{\text{j}}_{\text{F};1}$$

Dividing both sides of (126) by the total mass of the systems and defining the intensive thermodynamic property j associate to the extensive thermodynamic property J as:

(127) $$\text{j}=\frac{\text{J}({\text{n}}_{1},{\text{n}}_{\text{F}},{\text{x}}_{\text{f}3})}{{\text{n}}_{1}+{\text{n}}_{\text{F}}}$$

we obtain that:

(128) $$\text{j}={\text{x}}_{1}{\text{j}}_{1;\text{F}}+{\text{x}}_{\text{F}}{\text{j}}_{\text{F};1}$$

where x_F_ = n_F_/(n_1_ + n_F_), and x_1_ = 1 - x_F_. The Taylor’s expansion of first order of j = j(x_F_,x_f3_) with x_F_ close to zero is:

(129) $$\text{j}({\text{x}}_{\text{F}},{\text{x}}_{\text{f}3})=\text{j}(0,{\text{x}}_{\text{f}3})+{\left(\frac{\partial \text{j}(0,{\text{x}}_{\text{f}3})}{\partial {\text{x}}_{\text{F}}}\right)}_{{\text{x}}_{\text{f}3}}{\text{x}}_{\text{F}}$$

Using Equations (111) and (128):

(130) $$\text{j}(0,{\text{x}}_{\text{f}3})={\text{lim}}_{{\text{x}}_{\text{F}}\to 0}\;{\text{j}}_{1;\text{F}}({\text{x}}_{\text{F}},{\text{x}}_{\text{f}3})={\text{j}}_{1}$$

where j_1_ is the molar property of component 1 in the pure state. Using (128), we obtain:

(131) $${\left(\frac{\partial \text{j}({\text{x}}_{\text{F}},{\text{t}}_{\text{f}3})}{\partial {\text{x}}_{\text{F}}}\right)}_{{\text{x}}_{\text{f}3}}={\text{j}}_{\text{F};1}-{\text{j}}_{1;\text{F}}+\left[{\text{x}}_{1}{\left(\frac{\partial {\text{j}}_{1;\text{F}}}{\partial {\text{x}}_{\text{F}}}\right)}_{{\text{x}}_{\text{f}3}}+{\text{x}}_{\text{F}}{\left(\frac{\partial {\text{j}}_{\text{F};1}}{\partial {\text{x}}_{\text{F}}}\right)}_{{\text{x}}_{\text{f}3}}\right]$$

In our previous paper [6] we showed that:

(132) $${\text{x}}_{1}{\left(\frac{\partial {\text{j}}_{1;\text{F}}}{\partial {\text{x}}_{\text{F}}}\right)}_{{\text{x}}_{\text{f}3}}+{\text{x}}_{\text{F}}{\left(\frac{\partial {\text{j}}_{\text{F};1}}{\partial {\text{x}}_{\text{F}}}\right)}_{{\text{x}}_{\text{f}3}}=0$$

Taking the limit of x_F_ tending to zero in (131) and including (130) and (132) we obtain:

(133) $${\left(\frac{\partial \text{j}(0,{\text{x}}_{\text{f}3})}{\partial {\text{x}}_{\text{F}}}\right)}_{{\text{t}}_{\text{f}3}}={\text{lim}}_{{\text{x}}_{\text{F}}\to 0}\left[{\text{j}}_{\text{F};1}-{\text{j}}_{1;\text{F}}\right]={\text{j}}_{\text{F};1}^{\text{o}}-{\text{j}}_{1}$$

The substitution of (129) and (133) in (129) yields:

(134) $$\text{j}({\text{x}}_{\text{F}},{\text{x}}_{\text{f}3})={\text{j}}_{1}+\left({\text{j}}_{\text{F};1}^{\text{o}}({\text{x}}_{\text{f}3})-{\text{j}}_{1}\right){\text{x}}_{\text{F}}$$

or using Equation (114):

(135) $$\text{j}({\text{x}}_{\text{F}},{\text{x}}_{\text{f}3})={\text{j}}_{1}+\left({\text{x}}_{\text{f}2}{\text{j}}_{2;1,3}^{\Delta }({\text{x}}_{\text{f}3})+{\text{x}}_{\text{f}3}{\text{j}}_{3;1,2}^{\Delta }({\text{x}}_{\text{f}3})-{\text{j}}_{1}\right){\text{x}}_{\text{F}}$$

The effect of work in the high dilution region of j with respect to the variable x_F_ is to replace the partial properties as follows:

(136) $$\begin{array}{ll} {\text{j}}_{1;2,3}({\text{x}}_{\text{F}},{\text{x}}_{\text{f}3})= & {\text{j}}_{1;\text{F}}({\text{x}}_{\text{F}},{\text{x}}_{\text{f}3})\to {\text{j}}_{1}\\ & {\text{j}}_{2;1,3}({\text{x}}_{\text{F}},{\text{x}}_{\text{f}3})\to {\text{j}}_{2;1,3}^{\Delta }({\text{x}}_{\text{f}3})\\ & {\text{j}}_{3;1,2}({\text{x}}_{\text{F}},{\text{x}}_{\text{f}3})\to {\text{j}}_{3;1,2}^{\Delta }({\text{x}}_{\text{f}3})\\ & \;\;{\text{j}}_{\text{F};1}({\text{x}}_{\text{F}},{\text{x}}_{\text{f}3})\to {\text{j}}_{\text{F};1}^{\text{o}}({\text{x}}_{\text{f}3}) \end{array}$$

In the more simple case of a 2-component system, it is easy to see that Equation (134) takes the form:

(137) $$\text{j}({\text{x}}_{2})={\text{j}}_{1}+\left({\text{j}}_{2;1}^{\text{o}}-{\text{j}}_{1}\right){\text{x}}_{2}$$

In the high dilution region of j, the partial properties are replaced as:

(138) $$\begin{array}{l} {\text{j}}_{1;2}({\text{x}}_{2})\to {\text{j}}_{1}\\ {\text{j}}_{2;1}({\text{x}}_{2})\to {\text{j}}_{2;1}^{\text{o}} \end{array}$$

## Appendix 4. Basic Equations

In 3-component systems the enthalpy H is written as:

(139) $$\text{H}=\text{H}({\text{n}}_{1},{\text{n}}_{2},{\text{n}}_{3})$$

where its Euler’s equation takes the form:

(140) $$\text{H}={\text{n}}_{1}{\text{h}}_{1;2,3}+{\text{n}}_{2}{\text{h}}_{2;1,3}+{\text{n}}_{3}{\text{h}}_{3;1,2}$$

with h_1;2,3_, h_2;1,3_ and h_3;1,2_ being the partial molar properties of components 1, 2 and 3, respectively, defined as:

(141) $$\begin{array}{l} {\text{h}}_{1;2,3}={\left(\frac{\partial \text{H}}{\partial {\text{n}}_{1}}\right)}_{{\text{n}}_{2},{\text{n}}_{3}}\\ {\text{h}}_{2;1,3}={\left(\frac{\partial \text{H}}{\partial {\text{n}}_{2}}\right)}_{{\text{n}}_{1},{\text{n}}_{3}}\\ {\text{h}}_{3;1,2}={\left(\frac{\partial \text{H}}{\partial {\text{n}}_{3}}\right)}_{{\text{n}}_{2},{\text{n}}_{3}} \end{array}$$

Using the new variables c_F_, x_f3_ and V, the enthalpy takes the form:

(142) $$\text{H}=\text{H}({\text{c}}_{\text{F}},{\text{x}}_{\text{f}3},\text{V})$$

With the application of the Euler equation we have:

(143) $$\text{H}={\text{h}}_{\text{v}}({\text{c}}_{\text{F}},{\text{x}}_{\text{f}3})\text{V}$$

with:

(144) $${\text{h}}_{\text{v}}({\text{c}}_{\text{F}},{\text{x}}_{\text{f}3})={\left(\frac{\partial \text{H}}{\partial \text{V}}\right)}_{{\text{c}}_{\text{F}},{\text{x}}_{\text{f}3}}$$

Setting (140) and (143) equal to each other and, considering the following relationship between the variables n_1_, n_2_ and n_3_ and c_F_, x_f3_ and V:

(145) $$\begin{array}{l} {\text{n}}_{1}=\text{V}[\rho ({\text{c}}_{\text{F}},{\text{x}}_{\text{f}3})-{\text{c}}_{\text{F}}]\\ {\text{n}}_{2}={\text{Vc}}_{\text{F}}(1-{\text{x}}_{\text{f}3})\\ {\text{n}}_{3}={\text{Vc}}_{\text{F}}{\text{x}}_{\text{f}3} \end{array}$$

where ρ is the density of the system, we obtain:

(146) $${\text{h}}_{\text{v}}({\text{c}}_{\text{F}},{\text{x}}_{\text{f}3})=(\rho -{\text{c}}_{\text{F}}){\text{h}}_{1;2,3}+{\text{c}}_{\text{F}}(1-{\text{x}}_{\text{f}3}){\text{h}}_{2;1,3}+{\text{c}}_{\text{F}}{\text{x}}_{\text{f}3}{\text{h}}_{3;1,2}$$

If instead we consider the system as to be composed of component 1 and the fraction F (composed of the component 2 and 3), then (146) takes the form:

(147) $${\text{h}}_{\text{v}}({\text{c}}_{\text{F}},{\text{x}}_{\text{f}3})=(\rho -{\text{c}}_{\text{F}}){\text{h}}_{1;\text{F}}+{\text{c}}_{\text{F}}{\text{h}}_{\text{F};1}$$

Now we obtain an equation for (146) in the region of high dilution. For the specific partial enthalpies of 1, 2 and 3 we can make the following replacement by:

(148) $$\begin{array}{l} {\text{h}}_{1;\text{F}}({\text{x}}_{\text{F}},{\text{x}}_{\text{f}3})\to {\text{h}}_{1}\\ {\text{h}}_{\text{F};1}({\text{x}}_{\text{F}},{\text{x}}_{\text{f}3})\to {\text{h}}_{\text{F};1}^{\text{o}}({\text{x}}_{\text{f}3}) \end{array}$$

The density can be written in terms of the molar volume:

(149) $$\rho =\frac{1}{\text{v}}$$

Using Equation (134) it is possible to write an equation for the specific volume in the high dilution region:

(150) $$\text{v}({\text{x}}_{\text{F}},{\text{x}}_{\text{f}3})={\text{j}}_{1}+\left({\text{v}}_{\text{F};1}^{\text{o}}({\text{x}}_{\text{f}3})-{\text{v}}_{1}\right){\text{x}}_{\text{F}}$$

Substituting (150) in (149) and considering that c_F_ = x_F_ ρ:

(151) $$\rho ({\text{c}}_{\text{F}},{\text{x}}_{\text{f}3})=\rho_{1}+\left(1-\rho_{1}{\text{v}}_{\text{F};1}^{\text{o}}({\text{x}}_{\text{f}3})\right){\text{c}}_{\text{F}}$$

The equation for h_v_ in the high dilution region can be obtained by substituting Equations (151) and (148) in (147):

(152) $${\text{h}}_{\text{v}}({\text{c}}_{\text{F}},{\text{x}}_{\text{f}3})=\rho_{1}{\text{h}}_{1}+\left({\text{h}}_{\text{F};1}^{\text{o}}({\text{x}}_{\text{f}3})-\rho_{1}{\text{v}}_{\text{F};1}^{o}({\text{x}}_{\text{f}3})\right){\text{c}}_{\text{F}}$$

In the more simple case of a 2-component system the enthalpy can be written as:

(153) $$\text{H}({\text{c}}_{2},\text{V})={\text{h}}_{\text{v}}({\text{c}}_{2})\text{V}$$

where h_v_ can be written as:

(154) $${\text{h}}_{\text{v}}({\text{c}}_{2})={\text{h}}_{1;2}(\rho ({\text{c}}_{2})-{\text{c}}_{2})+{\text{h}}_{2;1}{\text{c}}_{2}$$

In the high dilution region Equation (154) takes the form:

(155) $${\text{h}}_{\text{v}}({\text{x}}_{2})=\rho_{1}{\text{h}}_{1}+\left({\text{h}}_{2;1}^{\text{o}}-\rho_{1}{\text{h}}_{1}{\text{v}}_{2;1}^{\text{o}}\right){\text{c}}_{2}$$
